# A meta-analysis of sutureless scleral-fixated intraocular lens versus retropupillary iris claw intraocular lens for the management of aphakia

**DOI:** 10.1038/s41598-023-49084-3

**Published:** 2024-01-24

**Authors:** Yu-Min Chang, Tzu-Heng Weng, Ming-Cheng Tai, Yi-Hao Chen, Cho-Hao Lee, Wei-Cheng Chang, Meng-Wei Hsieh, Ke-Hung Chien

**Affiliations:** 1 Department of Ophthalmology, Tri-Service General Hospital; and School of Medicine,, National Defense Medical Center, Number 325, Section 2, Chang-Gong Rd, Nei-Hu District, Taipei, 114 Taiwan, Republic of China; 2 Division of Hematology and Oncology Medicine, Department of Internal Medicine, Tri-Service General Hospital; and School of Medicine, National Defense Medical Center, Taipei, Taiwan; 3 Department of Ophthalmology, Taoyuan General Hospital, Ministry of Health and Welfare, Taoyuan, Taiwan; 4 Graduate Institute of Clinical Medicine, College of Medicine, Taipei Medical University, Taipei, Taiwan; 5 Department of Ophthalmology, Taoyuan Armed Forces General Hospital, Taoyuan, Taiwan

**Keywords:** Lens diseases, Vision disorders

## Abstract

This study compared the visual outcomes and complications between sutureless scleral-fixated intraocular lens and iris claw intraocular lens implantation in aphakia without adequate capsule and/or zonule support. Studies comparing the clinical outcomes of scleral-fixated intraocular lens and iris claw intraocular lens implantation published until April 2022 were retrieved from the PubMed, EMBASE, Cochrane Library, and Google Scholar databases. The outcomes included postoperative final visual acuity, surgical time, surgery-induced astigmatism, and complications. The weighted mean difference and odds ratio were calculated. Two randomized controlled trials and five cohort studies, including 244 and 290 eyes in the scleral-fixated intraocular lens group and iris claw group, respectively, were included. Scleral-fixated intraocular lens implantation results in a better postoperative final corrected distance visual acuity compared with iris claw intraocular lens implantation; however, it is more time-consuming. Scleral-fixated intraocular lens implantation seems to have lesser incidences of surgery-induced astigmatism. Furthermore, both procedures have a similar complication rate. Therefore, based on current best evidence, these two procedures should be considered according to patient’s conditions.

## Introduction

Phacoemulsification with posterior chamber intraocular lens (PCIOL) implantation is the most effective surgery for cataracts^1^. However, if the capsule and/or zonules are insufficient due to ocular trauma^2^, complicated surgery^3^, or hereditary diseases^4^, standard PCIOL implantation is contraindicated. Therefore, many methods have been developed for intraocular lens (IOL) implantation under insufficient capsule and/or zonule support conditions.

Anterior chamber intraocular lens (ACIOL) implantation was first developed in the 1950s and modified subsequently. ACIOL with flexible open-looped haptics has been used for aphakia without adequate capsule and/or zonule support^3,5^. However, ACIOL can cause complications, such as corneal edema, secondary glaucoma, uveitis, and hyphaemia^6^. Owing to its limitations and disadvantages, an increasing number of surgeons no longer use this technique.

Iris claw-fixated IOL was first introduced in 1972 to treat myopia^7^. Subsequently, several iris claw-fixated IOLs have been developed for use in aphakic eyes^8^. The iris claw-fixated IOL can be placed anterior to the iris or fixed posterior to the iris surface by the claws grasping the iris.

The scleral-fixated IOL can be placed in the sulcus or pars plana region located closest to the original lens. Hence, some surgeons prefer it over iris-fixated IOL. Suture-related problems have been reported for scleral-fixated IOL. In 2010, Scharioth et al.^9^ first described sutureless scleral-fixated intraocular lens (SSFIOL) surgery, which provided good visual results. Some surgeons have modified the method and used glue to secure IOL haptics in scleral tunnels^10^. In 2017, Yamane et al.^11^ proposed a new surgical technique in which the haptics of the IOL are cauterized and form a flange to fixate on the sclera. Since the Yamane technique does not require scleral flap creation, it saves time and has been widely adopted in recent years.

Many studies have compared the clinical outcomes and postoperative complications of SSFIOL and iris claw IOL implantation for aphakia without sufficient capsule and/or zonule support as these two methods have become more popular in recent years, but no consensus has been reached on which technique is better^12–18^. Hence, this study used data published until April 2022 to compare the clinical outcomes and complications of SSFIOL and iris claw IOL implantation in aphakia without adequate capsule and/or zonule support.

## Results

### Study selection

**Figure 1:**
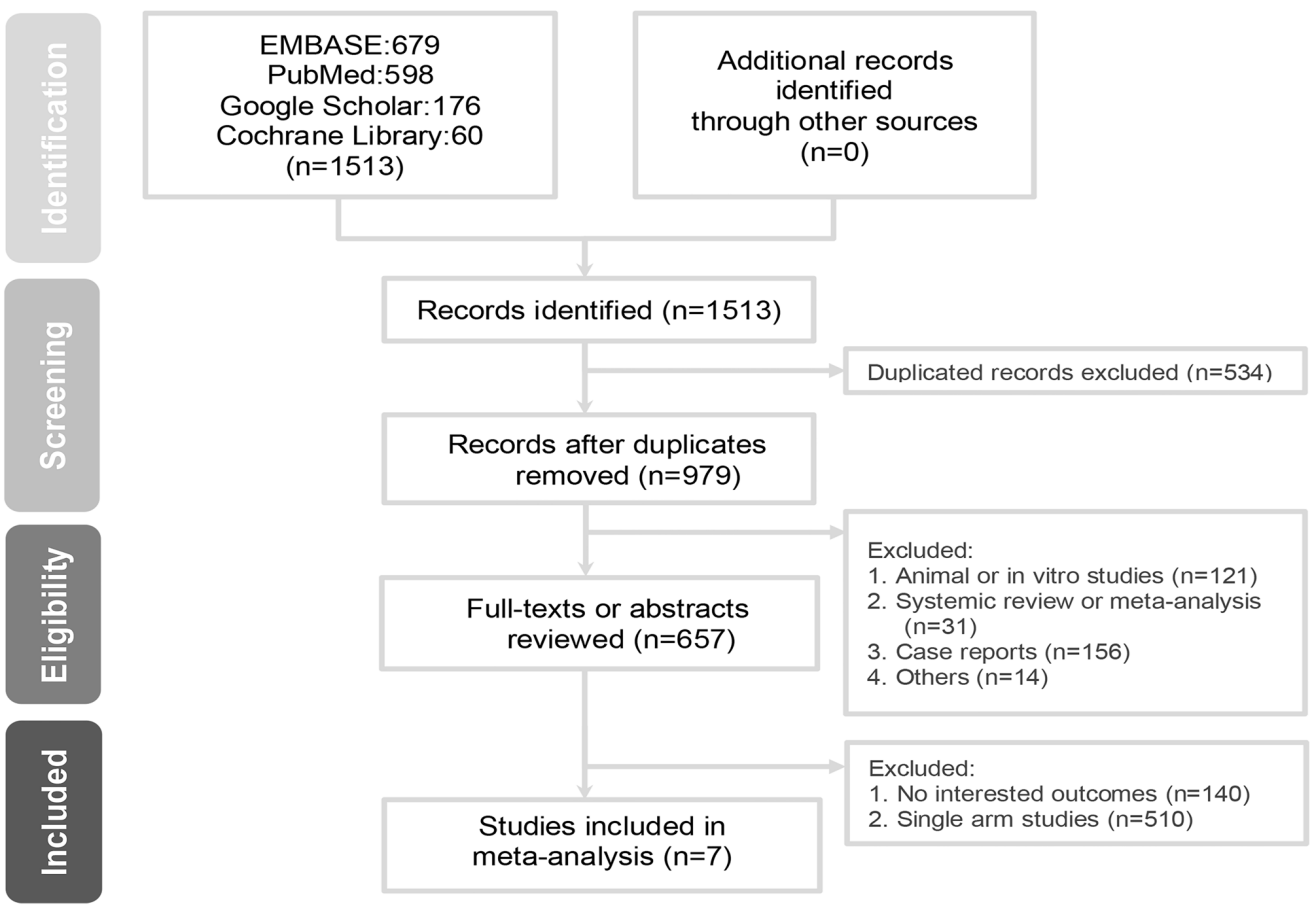
Flowchart of study selection process.

Figure 1 presents the flow chart of the study selection process. After reviewing 657 articles from electronic databases, seven articles were included according to the aforementioned criteria. Two randomized control trials (RCTs)^13,15^ and five cohort studies^12,14,16–18^ were identified, including six full-text articles and one conference abstract^16^. Thus, our meta-analysis included 534 eyes (244 and 290 eyes in the SSFIOL and iris-claw groups, respectively). The characteristics of the included studies are shown in Table 1. No significant differences in the pre-visual acuity (VA) values were found; however, two studies^16,18^ did not provide pre-VA values, although they reported similar VA values before surgery between the SSFIOL and iris claw groups. For the iris claw group, all studies reported retropupillary iris claw fixation. Furthermore, in these enrolled studies, the causes of aphakia include traumatic cataract^14,15,17^, complicated cataract followed by subluxated lens or IOL drop^12,14,18^, and congenital cataract^13^.

**Table 1:** Characteristics of the included studies.

Study	Trial design	Country	Eyes (N)	Pre-VA in the SSFIOL group (Mean ± SD) (logMAR)	Pre-VA in the iris claw group (Mean ± SD) (logMAR)	Follow-up duration (months)	Surgical technique in SSFIOL	Surgical technique in iris claw fixation
Saleh M et al.^17^	Cohort	France	26 (8 SSFIOL; 18 iris claw)	1.68 ± 1.15 (CDVA)	1.11 ± 1.13 (CDVA)	14	3-Piece IOL was exteriorised and tucked into scleral pockets	Retropupillary fixation
Madhivanan et al.^12^	Cohort	India	104 (56 SSFIOL; 48 iris claw)	1.74 ± 0.2 (UCDVA)	1.56 ± 0.3 (UCDVA)	12	3-Piece IOL was exteriorised and tucked into scleral pockets	Retropupillary fixation
Shuaib et al.^13^	RCT	Egypt	30 (15 SSFIOL; 15 iris claw)	0.23 ± 0.01 (CDVA)	0.21 ± 0.1 (CDVA)	6	3-Piece IOL was exteriorised and tucked into scleral pockets	Retropupillary fixation
Kelkar et al.^14^	Cohort	India	150 (60 SSFIOL; 90 iris claw)	1.48 ± 0.58 (UCDVA)	1.36 ± 0.64 (UCDVA)	12	3‑Piece IOL was externalised and the tip was heated with a thermal cautery to create a flange	Retropupillary fixation
Goyal et al.^15^	RCT	India	120 (60 SSFIOL; 60 iris claw)	1.66 ± 0.46 (UCDVA)	1.85 ± 0.61 (UCDVA)	12	3-Piece IOL was exteriorised and tucked into scleral pockets	Retropupillary fixation
Seknazi et al.^18^	Cohort	France	42 (20 SSFIOIL; 22 iris claw)	NA	NA	6	Sutureless trans-scleral plugs fixated lens was exteriorised and the plugs were tucked into scleral pockets	Retropupillary fixation
Bodin et al.^16^	Cohort	France	62 (25 SSFIOL; 37 iris claw)	NA	NA	3	Sutureless trans-scleral plugs fixated lens was exteriorised	Retropupillary fixation

*CDVA* corrected distance visual acuity, *IOL* intraocular lens, *N* number, *NA* not available, *RCT* randomized controlled trial, *SD* standard deviation, *SSFIOL* sutureless scleral-fixated intraocular lens, *UCDVA* uncorrected distance visual acuity.

### Quality of the individual studies

Supplementary Table S3 and Supplementary Figs. S1 and S2 show the evaluation of the bias risk of the enrolled studies. Two RCTs had low risk in random sequence generation^13,15^. Regarding allocation concealment, blinding of participants and personnel, the two RCTs revealed an unclear risk of bias; however, this might not have been a significant bias risk because our primary outcome, “postoperative final VA,” is an objective measurement. All RCTs had unclear blinding of the outcome assessment and did not have selective reporting or outcomes. However, for the cohort studies, we used the Newcastle–Ottawa scale to evaluate the bias risk: Four trials received eight stars and were identified as having the lower bias risk^12,14,17,18^, and one trial received four stars because only the abstract was available^16^.

### Primary outcomes

#### Postoperative final VA

**Figure 2:**
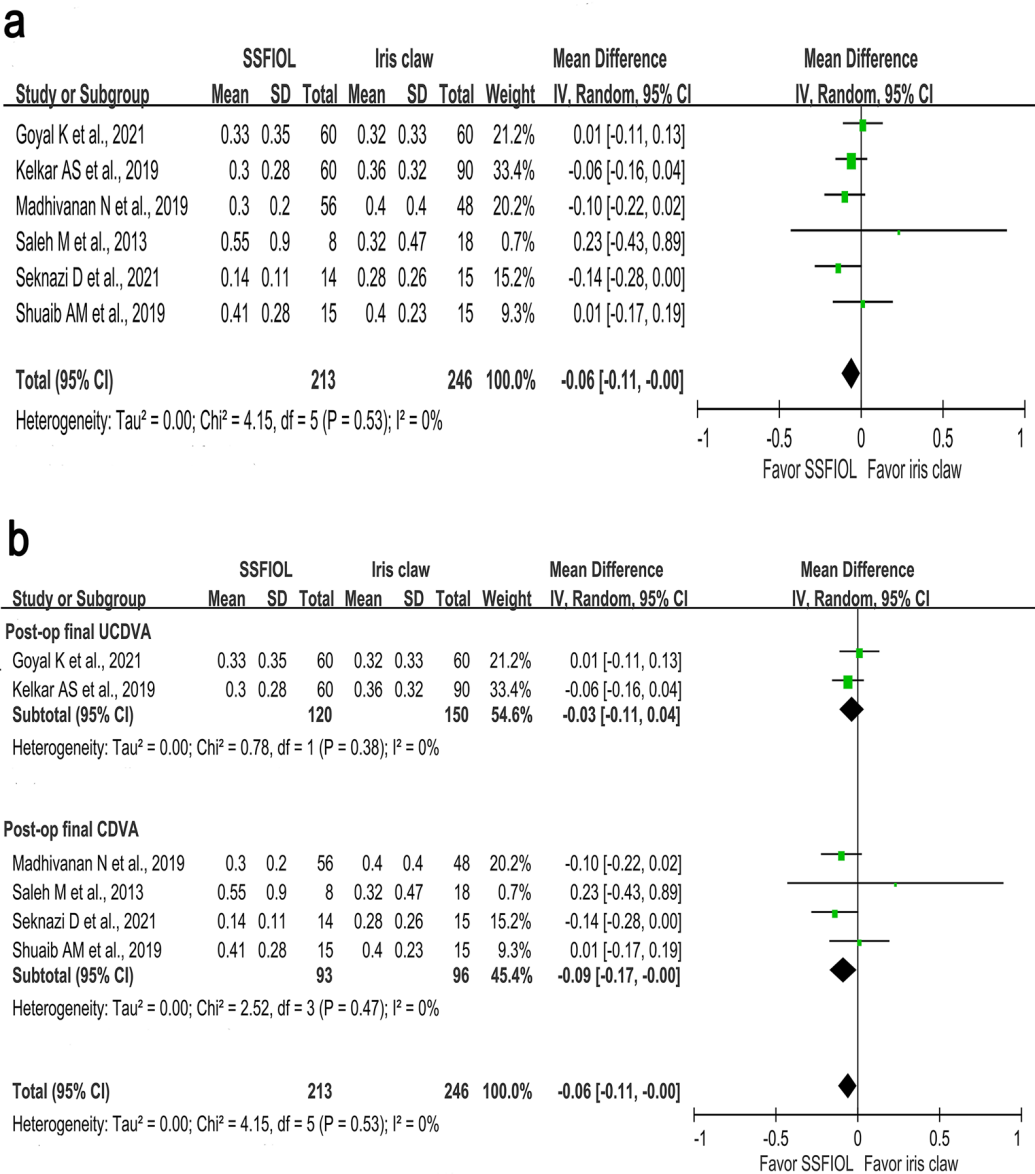
Forest plot of postoperative final VA comparing SSFIOL and iris claw IOL groups. (**a**) The forest plot reveals a trend of better postoperative final VA values in the SSFIOL group than in the iris claw IOL group, but the difference is not significant. (MD = -0.06; 95% CI − 0.11 to − 0.00; I^2^ = 0%, i = 0.05). (**b**) Subgroup analysis of postoperative final VA values. Postoperative final VA values are divided into postoperative final UCDVA and CDVA values in the SSFIOL and iris claw IOL groups. The subgroup analysis demonstrates the MD in the postoperative final UCDVA is − 0.03 (95% CI − 0.11 to 0.04; I^2^ = 0%, *P* = 0.4) and in the postoperative final CDVA was − 0.09 (95% CI − 0.17 to − 0.00; I = 0%, *P* = 0.04), respectively. The postoperative final CDVA is significantly better in the SSFIOL group than in the iris claw IOL group. CI, confidence interval; CDVA, corrected distance visual acuity; IOL, intraocular lens; SD, standard deviation; MD, mean deviation; SSFIOL, sutureless scleral-fixated intraocular lens; VA, visual acuity; UCDVA, uncorrected distance visual acuity.

Overall, 459 eyes (SSFIOL group, 213; iris claw group, 246) from two RCTs^13,15^ and four observational studies^12,14,17,18^ were included. The pooled data revealed that the SSFIOL group showed a trend towards better postoperative final VA values compared with those from the iris claw IOL group; however, the difference was not significant (MD =  − 0.06; 95% CI − 0.11 to − 0.00; I2 = 0%, *P* = 0.05) (Fig. 2A).

#### Subgroup analysis of primary outcome

The uncorrected distance visual acuity (UCDVA) and corrected distance visual acuity (CDVA) values were used by two^14,15^ and four^12,13,17,18^ studies, respectively, to represent the postoperative final VA values. Therefore, we aimed to determine if this reporting difference affected the primary outcome and conducted a subgroup analysis. The subgroup analysis demonstrated that the MD in postoperative final UCDVA values were − 0.03 (95% CI − 0.11 to 0.04; I^2^ = 0%, *P* = 0.4) for the postoperative final UCDVA group and − 0.09 (95% CI − 0.17 to − 0.00; I^2^ = 0%, *P* = 0.04) for the postoperative final CDVA group.

Therefore, if CDVA was used as the postoperative final VA, the SSFIOL group had a better VA outcome than the iris claw IOL group (Fig. 2B).

#### Publication bias

The funnel plots of the primary and secondary outcomes are presented in Supplementary Fig. S3. The Egger’s tests for primary and secondary outcomes were all above 0.05 and represented no significant publication bias. The funnel plot showed a visually symmetric distribution, and Egger’s test reported an insignificant *P* value = 0.74 for the primary outcome.

### Secondary outcomes

#### Surgical time

**Figure 3:**
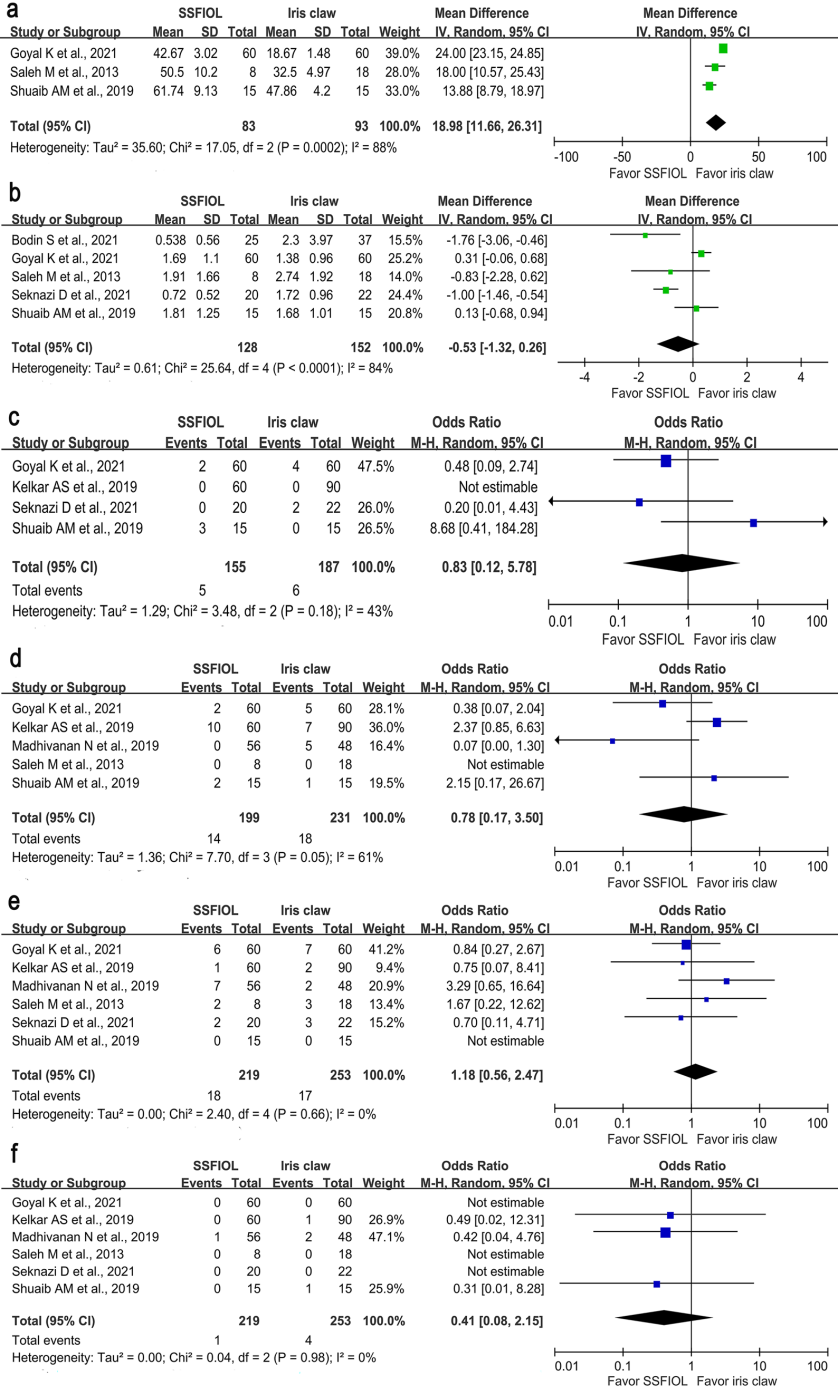
Forest plot of the secondary outcomes comparing the SSFIOL and iris claw IOL groups. (**a**) Surgical time. The iris claw IOL group has a significantly shorter surgical time than the SSFIOL group. (MD = 18.98; 95% CI 11.66–26.31; I^2^ = 88%, *P* < 0.001). (**b**) SIA. The extracted data reveals the SSFIOL group has a trend of lowering SIA, but the difference is not significant. (MD =  − 0.53; 95% CI − 1.32 to 0.26; I^2^ = 84%, *P* = 0.19). (**c**) IOL decentration/subluxation. The forest plot shows no statistically significant difference between the SSFIOL and iris claw IOL groups. (OR = 0.83; 95% CI 0.12–5.78; I^2^ = 43%, *P* = 0.85). (**d**) IOP elevation. The forest plot shows no significant difference in IOP elevation between the SSFIOL and iris claw IOL groups. (OR = 0.78; 95% CI 0.17–3.50; I^2^ = 61%, *P* = 0.75). (**e**) CME. The analysis of pooled data demonstrates there is no statistically significant difference between the SSFIOL and iris claw IOL groups. (OR = 1.18; 95% CI 0.56–2.47; I^2^ = 0%, *P* = 0.66). (**f**) Retinal detachment. The results show a trend of lower retinal detachment in the SSFIOL group, but not in the iris claw IOL group, but the difference is not significant. (OR = 0.41; 95% CI 0.08–2.15; I^2^ = 0%, *P* = 0.29). CI, confidence interval; CME, cystoid macular edema; IOL, intraocular lens; IOP, intraocular pressure; MD, mean deviation; OR, odds ratio; SIA, surgically induced astigmatism; SSFIOL, sutureless scleral-fixated intraocular lens.

In total, 176 eyes were included in the analyses, of which 83 were in the SSFIOL group, and 93 were in the iris claw group. The iris claw IOL group had a significantly shorter surgical time than the SSFIOL group. (MD = 18.98; 95% CI 11.66–26.31; I^2^ = 88%, *P* < 0.001) (Fig. 3A).

#### Surgery-induced astigmatism (SIA)

Overall, 206 and 288 eyes were included in the SSFIOL and iris claw groups, respectively. The incidence of SIA was lower in the SSFIOL group than that in the iris claw IOL group in three studies^16–18^. However, two studies^11,15^ reported that the iris claw IOL group had a lower incidence of SIA than the SSFIOL group. The extracted data revealed a trend toward a lower incidence of SIA in the SSFIOL group, but the difference was not significant (MD =  − 0.53; 95% CI − 1.32 to 0.26; I^2^ = 84%, *P* = 0.19) (Fig. 3B).

#### IOL decentration/subluxation

IOL decentration/subluxation is a common complication after IOL implantation without sufficient capsular support. Two RCTs^13,15^ and two cohort studies^14,18^ were analyzed. However, there was no statistically significant difference between the SSFIOL and iris claw IOL groups in the analysis (odds ratio [OR] = 0.83; 95% CI 0.12–5.78; I^2^ = 43%, *P* = 0.85) (Fig. 3C).

#### Intraocular pressure (IOP) elevation

Data were obtained from two RCTs^13,15^ and three cohort studies^12,14,17^, including 199 and 231 eyes in the SSFIOL and iris claw IOL groups, respectively. There was no significant difference in IOP elevation between the SSFIOL and iris claw IOL groups (OR = 0.78; 95% CI 0.17–3.50; I^2^ = 61%, *P* = 0.75) (Fig. 3D).

#### Cystoid macular edema (CME)

Two RCTs^13,15^ and four cohort studies^12,14,17,18^ included 219 and 253 eyes in the SSFIOL and iris claw IOL groups, respectively. However, analysis of the pooled data showed no statistically significant differences between the groups (OR 1.18; 95% CI 0.56–2.47; I^2^ = 0%, *P* = 0.66) (Fig. 3E).

#### Retinal detachment

Six studies (two RCTs^13,15^ and four cohort studies^12,14,17,18^) were included. The result showed a trend for lower retinal detachment in the SSFIOL group, but the difference was not significant (OR = 0.41; 95% CI 0.08–2.15; I^2^ = 0%, *P* = 0.29) (Fig. 3F).

## Discussion

Since the ACIOL has a high incidence of complications in treating aphakia without sufficient capsule and/or zonule support^6^, SSFIOL and iris claw IOL implantation have been the popular surgical techniques for treating aphakia without sufficient capsule and/or zonule support in recent years^12–18^. However, the visual outcomes and complication rate between SSFIOL and iris claw IOL implantation are controversial. Our study is the first meta-analysis to evaluate the clinical outcomes and complications of SSFIOL and iris claw IOL implantation in aphakia without adequate capsule and/or zonule support.

Postoperative VA is the most common concern after ophthalmic surgery. Our study demonstrated comparable VA results between the SSFIOL and iris claw IOL groups. The subgroup analysis indicated that the postoperative final CDVA was better in the SSFIOL group than that in the iris claw IOL group. This result contradicts a previous network meta-analysis^19^ that only included one study. Our meta-analysis included more recent studies (four studies), thereby providing more reliable results. Most postoperative visual impairments are likely more associated with postoperative complications and SIA. With the advent of new methods of scleral fixation and the invention of IOLs, surgeons become more proficient in scleral fixation techniques, it can not only reduce postoperative complications but also improve the vision of patients. Furthermore, there is also lesser SIA in the SSFIOL group in comparion with iris claw IOL group. The reduced postoperative complications and SIA may explain the better CDVA visual outcome in SSFIOL group. However, we still have to interpret the VA outcome with caution as there is not much of a difference in CDVA between the SSFIOL and iris claw IOL groups.

Surgical duration is another concern when choosing surgery. Many studies and analyses have demonstrated that iris claw IOL implantation requires a shorter surgical time than SSFIOL implantation^13,15,17,19,20^. Our analysis showed similar results in three studies. Different surgical techniques can impact the surgical duration, with a particularly diverse range of techniques in scleral fixation. The technique employed in the four articles^12,13,15,17^ involved 3-Piece IOL was exteriorised and tucked into scleral pockets, while the other two articles^16,18^ utilized a sutureless trans-scleral plugs fixated lens and exteriorised the plug under the scleral flap pockets. Whether using a 3-piece IOL or trans-scleral plugs fixated lens, a scleral flap requires suturing to secure the position of the haptics of IOL. In the final article^14^, the Yamane technique was employed to thermally the tip of 3‑Piece IOL to create a flange as a replacement for a scleral flap. On the other hand, the retropupillary iris claw IOL implantation, the surgical procedure generally involved making a corneal incision^12,13,17,18^ or sclerocorneal tunnel incision^14,15^ at 12 o'clock and creating two paracentesis wounds at 3 o'clock and 9 o'clock positions. The IOL was then inserted through the corneal incision, and instruments were used through the paracentesis wounds to secure the IOL beneath the iris. Finally, the corneal incision was sutured.

Although some studies have used other techniques to reduce the surgical time in SSFIOL implantation, such as using a flanged IOL to prevent the creation of scleral flaps^11,14^ and a sutureless scleral plug lens to promote the process of externalizing the haptics of the IOL becomes more convenient for grasping^18^, these studies did not compare the surgical time between the modified procedure in SSFIOL implantation and iris claw IOL implantation. Therefore, further analysis is required in the future.

SIA is the main reason for poor visual outcomes after cataract surgery and can be influenced by the corneal incision position and width^21^. Bodin et al.^16^ showed that SSFIOL implantation is associated with a lower incidence of SIA than iris claw IOL implantation because SSFIOL implantation only requires a 3-mm, instead of a 5–6-mm, corneal incision. Although SSFIOL implantation can result in smaller corneal incision wounds, scleral flap creation may also influence corneal astigmatism. Using pooled data for analysis, we found a trend for lesser SIA in the SSFIOL group, but this was not significant.

Postoperative complications were rare in our analysis, and there was no statistically significant difference between the groups. IOL decentration/subluxation is a common complication after IOL implantation. Shuaib et al.^13^ reported that IOL decentration was more common in SSFIOL because of haptic slippage and easy deformation of the 3-piece IOL during exteriorization. Conversely, two studies reported that iris claw IOLs were more easily decentred due to poor iris enclavation^15,18^. The inconclusive results may be related to surgeons’ skills and patient conditions.

IOP elevation after IOL implantation has been reported in many studies^22,23^, and it is also commonly caused by postoperative steroid usage, anterior chamber inflammation, and pigment dispersion. Many studies revealed transient IOP elevation after surgery and these patients were treated with medication^12,14,15^. Our analysis revealed there was no significant difference in IOP elevation between the SSFIOL and iris claw IOL groups.

Postsurgical CME is another common complication of cataract surgeries^24^. Its etiology is not clearly understood, but vitreous traction and inflammatory conditions in the eyes have been proposed as the causative agents^24^. Madhivanan et al.^12^ discovered a higher incidence of CME in the SSFIOL group than that in the iris claw IOL group, suggesting that the use of triamcinolone-assisted vitrectomy in the iris claw IOL group reduced CME incidences. However, Liang et al.^25^ conducted a meta-analysis and found that iris claw IOLs in the anterior chamber had a higher incidence of CME compared with retropupillary implantations of iris claw IOLs, potentially caused by different grades of inflammation or pigment dispersion between the anterior and posterior surfaces of the iris. In our analysis, all studies used retropupillary implantation for iris claw IOLs, except one study that did not reveal this information^16^. Our analysis demonstrated no significant differences between the groups.

Regarding retinal detachment after cataract surgery, the most acceptable etiology is vitreous body destabilization^26^. Since our analysis included patients with aphakia without adequate capsule and/or zonule support, almost all patients underwent posterior vitrectomy^12,14,15,17,18^, which can reduce the vitreous traction force on the retina. We consider this to be the reason for the small number of cases in our analysis. There is only one study focused on pediatric patients, primarily involving anterior vitrectomy^13^.

Our meta-analysis had some limitations. First, we only included seven studies (two RCTs and five cohort studies), which could have influenced the reliability and validity of our study. Second, SSFIOL implantations require more surgical time and techniques than iris claw IOL implantation. This could have influenced the surgeons’ choice, causing selection bias. Furthermore, various articles presented slight differences in surgical techniques, and there was also diversity in the brands of IOLs used. These variations may impact the duration of the surgery and the various outcomes we assessed. Third, to enlarge the sample size, we included a study with pediatric patients, postoperative inflammation and complications differed slightly from those in adults, which can also affect the assessment of our results. Such as the IOP elevation in pediatric patients, this condition can originally related to microspherophakia which is known to be associated with glaucoma. Fourth, the causes of aphakia in the included studies varied, and insufficient preoperative patient data were obtained. Fifth, only common complications were included in our analysis. Lastly, the follow-up duration varied between studies, which could have affected our outcome analyses.

In conclusion, our meta-analysis revealed that eyes with SSFIOL implantations had better postoperative final CDVA values and lower incidences of SIA than those of eyes with iris claw IOLs, but the difference was small. However, iris claw IOL implantation involves a significantly shorter surgical time. These two procedures had similar complication rates in our analysis. Since both procedures have pros and cons, we suggest surgeons choose the appropriate surgery in patients with aphakia without sufficient capsule and/or zonule support according to the patient’s condition. We expect more RCTs will be conducted in the future to confirm our conclusions.

## Methods

### Search strategy

We searched the databases of PubMed, EMBASE, Cochrane Library, and Google Scholar comprehensively using the following keywords: “iris claw” OR “iris-fix” OR “iris-clip” OR “iris suture” OR “artisan” OR “Verisyse” combined with “intrascleral fix” OR “sutureless intrascleral fix” OR “sutureless scleral fix” OR “sutureless trans-scleral fix” OR “Yamane” OR “flanged intrascleral fix” OR “intrascleral haptic fixation” without language restriction to obtain studies published through April 2022. To obtain complete data, conference material, abstracts, and reference lists cited in previous studies were manually searched. The search strategy is presented in Supplementary Table S1.

### Inclusion and exclusion criteria

This meta-analysis investigated patients with aphakia without sufficient capsule and/or zonule support and compared the clinical outcomes and complications of the SSFIOL and iris claw-fixated IOL groups. We included studies published before April 2022 that satisfied our inclusion criteria.

The inclusion criteria were as follows: (1) study design: RCTs, comparative cohort studies, and case–control studies; (2) population: patients with aphakia without sufficient capsule and/or zonule support; (3) intervention: comparison of SSFIOL with iris-claw fixated IOL; and (4) outcomes: evaluation of one of the following clinical outcomes: postoperative final VA, postoperative final CDVA, surgical time, SIA, and postoperative complications. We included full-text articles and conference abstracts that provided useful and sufficient information. The primary reason for including the abstract is the limited number of included articles, and since this article contains the results we wanted to assess, we decided to include it.

The exclusion criteria were as follows: (1) animal or in vitro studies; (2) systemic reviews and meta-analyses; (3) studies not involving the clinical outcomes per our inclusion criteria; and (4) single-arm studies.

The Preferred Reporting Items for Systematic Reviews and Meta-Analyses checklist is presented in Supplementary Table S2.

### Data extraction

Two authors independently conducted abstract screening and data extraction (YMC and KHC). The extracted data included study design, publication year, region, number of eyes, patient characteristics (mean age, preoperative VA, and sex), follow-up duration, surgical techniques, postoperative VA, surgical time, SIA, and postoperative complications. Conflicts between the two reviewers were resolved through discussion or by a senior reviewer.

### Quality assessment

Two authors independently performed a quality assessment of RCTs according to the Cochrane Collaboration Reviewers’ Handbook for Systematic Reviews of Interventions^27^. The scores ranged from 0 (high bias) to 7 (low bias). The Newcastle–Ottawa scale was used to evaluate the quality of the cohort studies^28^. The quality assessment comprised three broad perspectives: selection of the study groups, comparability of the groups, and ascertainment of the exposure or outcome of interest for cohort studies. The star system was used to evaluate the risk of bias, with each star representing a low bias. The study could have 0 (lowest quality) to 9 (highest quality) stars. If the two reviewers had disagreements regarding the quality assessment, a discussion was had until a consensus was reached or the senior reviewer made the final decision.

### Outcome evaluations

The postoperative final VA was our primary outcome. We also conducted a subgroup analysis of our primary outcome to determine if there was a difference between UCDVA and CDVA. The surgical time and SIA were evaluated as secondary outcomes. Postoperative complications are important parameters in any surgery; therefore, we investigated IOL decentration/subluxation, IOP elevation, CME, and retinal detachment as secondary outcomes to determine the safety of these two surgeries.

### Statistical analyses

The demographic characteristics of the included studies are presented as mean ± standard deviation or numbers according to the parameters. The weighted MD was calculated for continuous variables, and the OR was used for dichotomous variables. We used Review Manager (RevMan v5.3 2014) to conduct the analyses. Statistical significance was defined as a *P* value < 0.05. Egger’s regression and funnel plots were used to investigate publication bias. I^2^ represents the heterogeneity of studies, and I^2^ values < 25% were regarded as having low heterogeneity^29^. We used the Mantel–Haenszel random-effects model when I^2^ > 50% and even when I^2^ < 50% due to the small number of enrolled studies.

### Supplementary Information

Supplementary Information.

## Data Availability

The corresponding author (KHC) had full access to all the data in the study and takes responsibility for the integrity of the data and the accuracy of the data analysis. The data and materials used in this study can be obtained from the corresponding author (KHC) upon request.
