# Supplementary material for: A meta-analysis of sutureless scleral-fixated intraocular lens versus retropupillary iris claw intraocular lens for the management of aphakia
Source: Sci Rep. 2024 Jan 24;14:2044. doi: 10.1038/s41598-023-49084-3 (PMC10808084; doi:10.1038/s41598-023-49084-3)
Supplement: Supplementary file 1 — Supplementary Information. [file 41598_2023_49084_MOESM1_ESM.docx]

**Supplementary Information**

**A meta-analysis of sutureless scleral-fixated intraocular lens versus retropupillary iris claw intraocular lens for the management of aphakia**

Contents

Supplementary Table S1. Search strategies and detailed records

Supplementary Table S2. PRISMA checklist and search strategies and detailed records

Supplementary Table S3. Newcastle–Ottawa quality assessment scale for cohort studies

Supplementary Figure S1. Risk of bias summary

Supplementary Figure S2. Risk of bias bar graph

Supplementary Figure S3. Funnel plots of primary and secondary outcomes

**Supplementary Table S1.** Search strategies and detailed records

**Search strategies**

| **Relevant text of Population**   1. Lens Implantations, Intraocular 2. Implantation, Intraocular Lens 3. Intraocular Lens Implantation 4. Intraocular Lens Implantations 5. Implantations, Intraocular Lens 6. Cataract Extraction 7. Extraction, Cataract 8. Extractions, Cataract   A= #1 or #2 or #3 or #4 or #5 or #6 or #7 or #8 | **Relevant text of intervention**   1. Iris-claw 2. Iris-fix 3. Iris-clip 4. Iris suture 5. Artisan 6. Verisyse 7. Intrascleral fix 8. Sutureless intrascleral fix 9. Sutureless scleral fix 10. Sutureless transscleral fix 11. Flanged intrascleral fix 12. Intrascleral haptic fixation 13. Yamane   B = #1 or #2 or #3 or #4 or #5 or #6 or #7 or #8 or #9 or #10 or #11 or #12 or #13  *. All search keyword with [Mesh Terms] or [All Fields] |
| --- | --- |

Web sites and uniform resource locator:

**PUBMED**: <http://www.ncbi.nlm.nih.gov/pubmed>

**EMBASE**: https://www.embase.com

**COCHRANE CENTRAL**: https://www.cochrane.com

**Search details**

| PubMed data base | | |
| --- | --- | --- |
| Population | |  |
| A | (((((((Lens Implantations, Intraocular [Title/Abstract]) OR Implantation, Intraocular Lens [Title/Abstract]) OR Intraocular Lens Implantation [Title/Abstract]) OR Intraocular Lens Implantations [Title/Abstract]) OR Implantations, Intraocular Lens [Title/Abstract])) OR (Cataract Extraction[Title/Abstract])) OR (Extraction, Cataract[Title/Abstract])) OR (Extractions, Cataract[Title/Abstract]) | **33872** |
| Intervention | |  |
| B | Intervention  ((((((((((((Iris-claw[Title/Abstract]) OR (Iris-fix[Title/Abstract])) OR (Iris-clip[Title/Abstract])) OR (Iris suture[Title/Abstract])) OR (Artisan[Title/Abstract])) OR (Verisyse[Title/Abstract])) OR (Intrascleral fix[Title/Abstract])) OR (Sutureless intrascleral fix[Title/Abstract])) OR (Sutureless scleral fix[Title/Abstract])) OR (Sutureless transscleral fix[Title/Abstract])) OR (Flanged intrascleral fix[Title/Abstract])) OR (Intrascleral haptic fixation[Title/Abstract])) OR (Yamane[Title/Abstract]) | **13977** |
| A & B | Population & Intervention  ((((((((Lens Implantations, Intraocular [Title/Abstract]) OR Implantation, Intraocular Lens [Title/Abstract]) OR Intraocular Lens Implantation [Title/Abstract]) OR Intraocular Lens Implantations [Title/Abstract]) OR Implantations, Intraocular Lens [Title/Abstract])) OR (Cataract Extraction[Title/Abstract])) OR (Extraction, Cataract[Title/Abstract])) OR (Extractions, Cataract[Title/Abstract])) AND (((((((((((((Iris-claw[Title/Abstract]) OR (Iris-fix[Title/Abstract])) OR (Iris-clip[Title/Abstract])) OR (Iris suture[Title/Abstract])) OR (Artisan[Title/Abstract])) OR (Verisyse[Title/Abstract])) OR (Intrascleral fix[Title/Abstract])) OR (Sutureless intrascleral fix[Title/Abstract])) OR (Sutureless scleral fix[Title/Abstract])) OR (Sutureless transscleral fix[Title/Abstract])) OR (Flanged intrascleral fix[Title/Abstract])) OR (Intrascleral haptic fixation[Title/Abstract])) OR (Yamane[Title/Abstract])) | **598** |
| Cochrane data base | |  |
| Population | |  |
| A | Population  (([mh Lens Implantations, Intraocular] OR Lens Implantation, Intraocular:ti,ab) OR [mh Cataract Extraction] OR Cataract Extraction:ti,ab | **5083** |
| Intervention | |  |
| B | Intervention  [mh "Iris-claw"] OR Iris-claw:ti,ab OR [mh Iris-fix] OR Iris-fix:ti,ab OR [mh Iris-clip] OR Iris-clip:ti,ab OR [mh Iris suture] OR Iris suture:ti,ab OR [mh Artisan] OR Artisan:ti,ab OR [mh Verisyse] OR Verisyse:ti,ab [mh Intrascleral fix] OR Intrascleral fix:ti,ab OR [mh Sutureless intrascleral fix] OR Sutureless intrascleral fix:ti,ab OR [mh Sutureless scleral fix] OR Sutureless scleral fix:ti,ab OR [mh Sutureless transscleral fix] OR Sutureless transscleral fix:ti,ab OR [mh Flanged intrascleral fix] OR Flanged intrascleral fix:ti,ab OR [mh Intrascleral haptic fixation] OR Intrascleral haptic fixation:ti,ab OR [mh Yamane] OR Yamane:ti,ab | **257** |
| A & B | Population & Intervention  ((([mh Lens Implantations, Intraocular] OR Lens Implantation, Intraocular:ti,ab) AND [mh Intraocular Lens Implantation] OR Intraocular Lens Implantation:ti,ab OR [mh Sulcus] OR Sulcus:ti,ab) AND ([mh " Optic capture] OR Optic capture:ti,ab) AND ([mh "Iris-claw"] OR Iris-claw:ti,ab OR [mh Iris-fix] OR Iris-fix:ti,ab OR [mh Iris-clip] OR Iris-clip:ti,ab OR [mh Iris suture] OR Iris suture:ti,ab OR [mh Artisan] OR Artisan:ti,ab OR [mh Verisyse] OR Verisyse:ti,ab [mh Intrascleral fix] OR Intrascleral fix:ti,ab OR [mh Sutureless intrascleral fix] OR Sutureless intrascleral fix:ti,ab OR [mh Sutureless scleral fix] OR Sutureless scleral fix:ti,ab OR [mh Sutureless transscleral fix] OR Sutureless transscleral fix:ti,ab OR [mh Flanged intrascleral fix] OR Flanged intrascleral fix:ti,ab OR [mh Intrascleral haptic fixation] OR Intrascleral haptic fixation:ti,ab OR [mh Yamane] OR Yamane:ti,ab ))) | **60** |
| Embase data base | |  |
| Population | |  |
| A | Population  'cataract extraction'/exp OR 'cataract extraction' OR 'intraocular lens implantation'/exp OR 'intraocular lens implantation' | **60387** |
| Intervention | |  |
| B | Intervention  'iris-claw' OR 'iris-fix' OR 'iris-clip' OR 'iris suture' OR 'artisan'/exp OR 'artisan' OR 'verisyse'/exp OR 'verisyse' OR 'intrascleral fix' OR 'sutureless intrascleral fix' OR 'sutureless scleral fix' OR 'sutureless transscleral fix' OR 'flanged intrascleral fix' OR 'intrascleral haptic fixation' OR 'yamane' | **10477** |
| A & B | Population & Intervention  'cataract extraction'/exp OR 'cataract extraction' OR 'intraocular lens implantation'/exp OR 'intraocular lens implantation' AND 'iris-claw' OR 'iris-fix' OR 'iris-clip' OR 'iris suture' OR 'artisan'/exp OR 'artisan' OR 'verisyse'/exp OR 'verisyse' OR 'intrascleral fix' OR 'sutureless intrascleral fix' OR 'sutureless scleral fix' OR 'sutureless transscleral fix' OR 'flanged intrascleral fix' OR 'intrascleral haptic fixation' OR 'yamane | **679** |
| **Google** | |  |
|  | Intraocular Lens Implantation + Iris-claw + Sutureless scleral fix | **555** |
|  | Intraocular Lens Implantation + Iris-claw + Sutureless scleral fix + Human studies | **176** |

**Supplementary Table S2.** PRISMA checklist

| **Section/topic** | **Item No** | **Checklist item** | **Reported on page No** |
| --- | --- | --- | --- |
| **Title** | | | |
| Title | 1 | Identify the report as a systematic review, meta-analysis, or both | 1 |
| **Abstract** | | | |
| Structured summary | 2 | Provide a structured summary including, as applicable, background, objectives, data sources, study eligibility criteria, participants, interventions, study appraisal and synthesis methods, results, limitations, conclusions and implications of key findings, systematic review registration number | Abstract 3 |
| **Introduction** | | | |
| Rationale | 3 | Describe the rationale for the review in the context of what is already known | 4 |
| Objectives | 4 | Provide an explicit statement of questions being addressed with reference to participants, interventions, comparisons, outcomes, and study design (PICOS) | 5 |
| **Methods** | | | |
| Protocol and registration | 5 | Indicate if a review protocol exists, if and where it can be accessed (such as web address), and, if available, provide registration information including registration number | None |
| Eligibility criteria | 6 | Specify study characteristics (such as PICOS, length of follow-up) and report characteristics (such as years considered, language, publication status) used as criteria for eligibility and giving rationale | 15-16 |
| Information sources | 7 | Describe all information sources (such as databases with dates of coverage, contact with study authors to identify additional studies) in the search and date last searched | 16 |
| Search | 8 | Present full electronic search strategy for at least one database, including any limits used, such that it could be repeated | 16 |
| Study selection | 9 | State the process for selecting studies (that is, screening, eligibility, included in systematic review, and, if applicable, included in the meta-analysis) | 16-17 |
| Data collection process | 10 | Describe method of data extraction from reports (such as piloted forms, independently, in duplicate) and any processes for obtaining and confirming data from investigators | 16-17 |
| Data items | 11 | List and define all variables for which data were sought (such as PICOS, funding sources) and any assumptions and simplifications made | 16-17 |
| Risk of bias in individual studies | 12 | Describe methods used for assessing risk of bias of individual studies (including specification of whether this was done at the study or outcome level), and how this information is to be used in any data synthesis | 17 |
| Summary measures | 13 | State the principal summary measures (such as risk ratio, difference in means). | 18-19 |
| Synthesis of results | 14 | Describe the methods of handling data and combining results of studies, if done, including measures of consistency (such as I^2^ statistic) for each meta-analysis | 18 |
| Risk of bias across studies | 15 | Specify any assessment of risk of bias that may affect the cumulative evidence (such as publication bias, selective reporting within studies) | 17 |
| Additional analyses | 16 | Describe methods of additional analyses (such as sensitivity or subgroup analyses, meta-regression), if done, indicating which were pre-specified | 18-19 |
| **Results** | | | |
| Study selection | 17 | Give numbers of studies screened, assessed for eligibility, and included in the review, with reasons for exclusions at each stage, ideally with a flow diagram | 5, Figure 1 |
| Study characteristics | 18 | For each study, present characteristics for which data were extracted (such as study size, PICOS, follow-up period) and provide the citations | 7, Table 1 |
| Risk of bias within studies | 19 | Present data on risk of bias of each study and, if available, any outcome-level assessment (see item 12). | 6-7, Supplementary Table S3, figure S1,S2 |
| Results of individual studies | 20 | For all outcomes considered (benefits or harms), present for each study (a) simple summary data for each intervention group and (b) effect estimates and confidence intervals, ideally with a forest plot | 11-15 |
| Synthesis of results | 21 | Present results of each meta-analysis done, including confidence intervals and measures of consistency | 7-11, Figures 2-3 |
| Risk of bias across studies | 22 | Present results of any assessment of risk of bias across studies (see item 15) | 6-7, Supplementary Table S3,figure S1,S2 |
| Additional analysis | 23 | Give results of additional analyses, if done (such as sensitivity or subgroup analyses, meta-regression) (see item 16) | N/A |
| **Discussion** | | | |
| Summary of evidence | 24 | Summarize the main findings including the strength of evidence for each main outcome; consider their relevance to key groups (such as health care providers, users, and policy makers) | 11-14 |
| Limitations | 25 | Discuss limitations at study and outcome level (such as risk of bias), and at review level (such as incomplete retrieval of identified research, reporting bias) | 14 |
| Conclusions | 26 | Provide a general interpretation of the results in the context of other evidence, and implications for future research | 15 |
| **Funding** | | | |
| Funding | 27 | Describe sources of funding for the systematic review and other support (such as supply of data) and role of funders for the systematic review | 2 |

**Supplementary Table S3.** **Newcastle–Ottawa quality assessment scale for cohort studies**

Note: A study can be awarded a maximum of one star for each numbered item within the Selection and Outcome categories. A maximum of two stars can be given for comparability

| **Study** | **Representativeness of the exposed cohort** | **Selection of the non- exposed cohort** | **Ascertainment of exposure** | **Demonstration that outcome of interest was not present at the start of the study** | **Comparability of cohorts on the basis of the design or analysis** | **Assessment of outcome** | **Was follow-up long enough for outcomes to occur** | **Adequacy of follow-up of cohorts** | **Scores** |
| --- | --- | --- | --- | --- | --- | --- | --- | --- | --- |
| *Saleh M et al.,*  *2013* | ★ | ★ | ★ | ★ | ★ | ★ | ★ | ★ | 8 |
| *Madhivanan N et al.,*  *2019* | ★ | ★ | ★ | ★ | ★ | ★ | ★ | ★ | 8 |
| *Kelkar AS et al.,*  *2019* | ★ | ★ | ★ | ★ | ★ | ★ | ★ | ★ | 8 |
| *Bodin S et al., 2021* | ★ | ★ |  | ★ |  |  | ★ |  | 4 |
| *Seknazi D et al., 2021* | ★ | ★ | ★ | ★ | ★ | ★ | ★ | ★ | 8 |

**Supplementary Figure S1.** Risk of bias summary


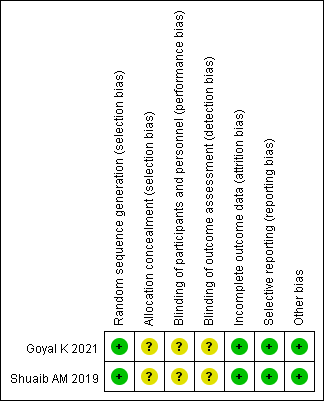


**Supplementary Figure S2.** Risk of bias bar graph


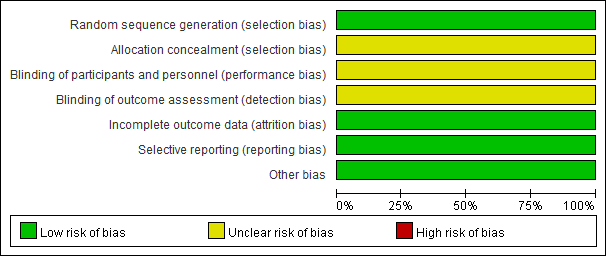


**Supplementary Figure S3.** Funnel plots of primary and secondary outcomes

The Egger’s test value for primary and secondary outcomes is above 0.05 and represents no significant publication bias

| Post-op final VA | Surgical time | Surgery-induced astigmatism |
| --- | --- | --- |
| 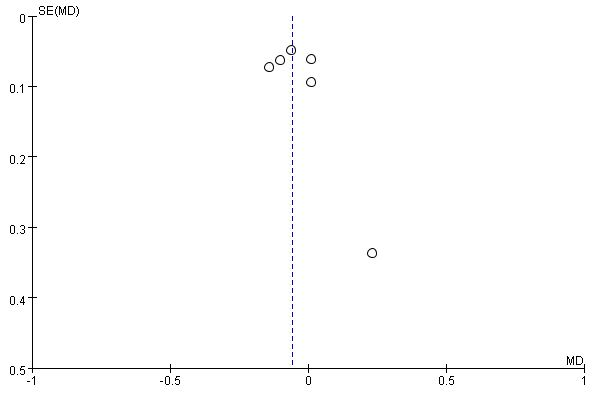 | 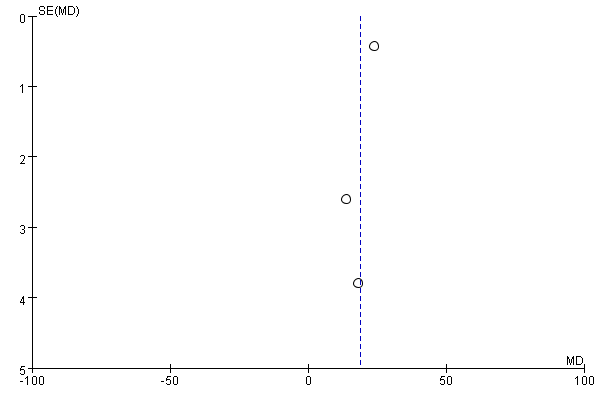 | 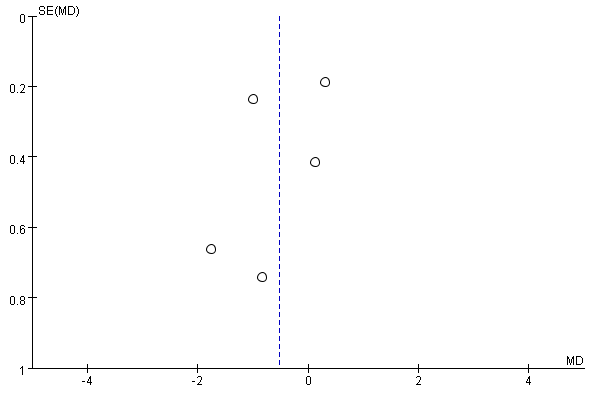 |
| IOL decentration/subluxation | IOP elevation | Cystoid macular edema |
| 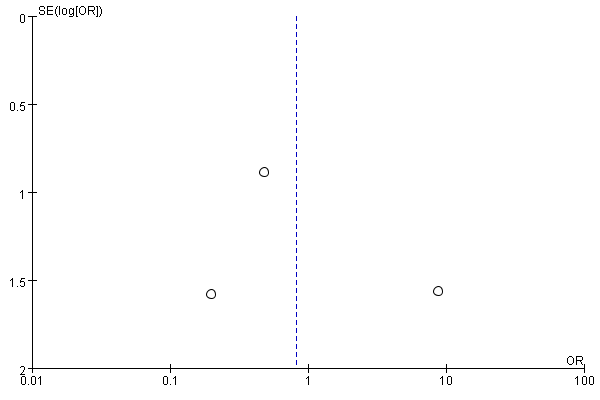 | 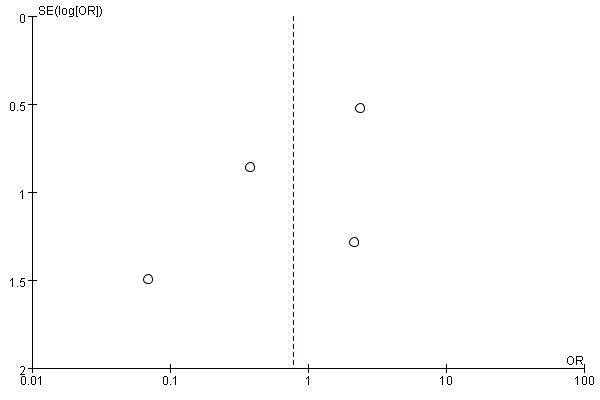 | 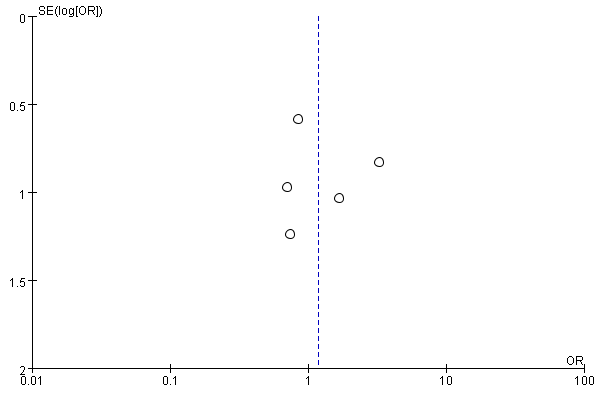 |
| Retinal detachment |  |  |
| 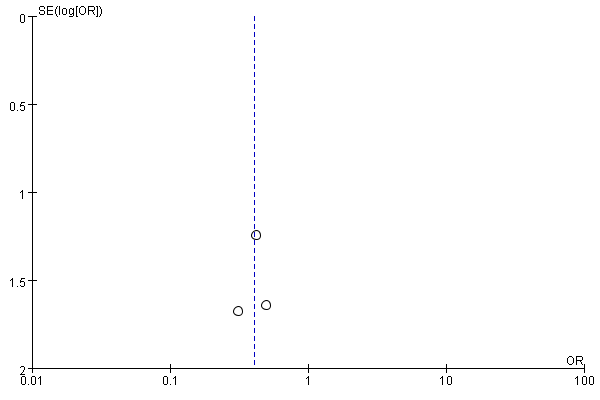 |  |  |

Post-op, post-operative; VA, visual acuity; IOL, intraocular lens; IOP, intraocular pressure
